# Differentially expressed autophagy-related genes are potential prognostic and diagnostic biomarkers in clear-cell renal cell carcinoma

**DOI:** 10.18632/aging.102368

**Published:** 2019-10-17

**Authors:** Bangbei Wan, Bo Liu, Gang Yu, Yuan Huang, Cai Lv

**Affiliations:** 1 Department of Urology, Central South University Xiangya School of Medicine Affiliated Haikou Hospital, Haikou 570208, Hainan, China; 2 Laboratory of Developmental Cell Biology and Disease, School of Ophthalmology and Optometry and Eye Hospital, Wenzhou Medical University, Wenzhou 325003, China; 3 Department of Neurology, Central South University Xiangya School of Medicine Affiliated Haikou Hospital, Haikou 570208, Hainan, China

**Keywords:** autophagy, ccRCC, TCGA database, prognosis, autophagy-related genes

## Abstract

We examined the role of differentially expressed autophagy-related genes (DEARGs) in clear cell Renal Cell Carcinoma (ccRCC) using high-throughput RNA-seq data from The Cancer Genome Atlas (TCGA). Cox regression analyses showed that 5 DEARGs (*PRKCQ*, *BID*, *BAG1*, *BIRC5*, and *ATG16L2*) correlated with overall survival (OS) and 4 DEARGs (*EIF4EBP1*, *BAG1*, *ATG9B*, and *BIRC5*) correlated with disease-free survival (DFS) in ccRCC patients. Multivariate Cox regression analysis using the OS and DFS prognostic risk models showed that expression of the nine DEARGs accurately and independently predicted the risk of disease recurrence or progression in ccRCC patients (area under curve or AUC values > 0.70; all *p* < 0.05). Moreover, the DEARGs accurately distinguished healthy individuals from ccRCC patients based on receiver operated characteristic (ROC) analyses (area under curve or AUC values > 0.60), suggesting their potential as diagnostic biomarkers for ccRCC. The expression of DEARGs also correlated with the drug sensitivity of ccRCC cell lines. The ccRCC cell lines were significantly sensitive to Sepantronium bromide, a drug that targets *BIRC5*. This makes *BIRC5* a potential therapeutic target for ccRCC. Our study thus demonstrates that DEARGs are potential diagnostic and prognostic biomarkers and therapeutic targets in ccRCC.

## INTRODUCTION

Clear cell renal cell carcinoma (ccRCC) accounts for 70–80% of cases of renal cell carcinoma (RCC), which is one of the most common malignant tumors of the urinary system [1, 2]. Currently, computed tomography (CT) is the main imaging technique used for the diagnosis and staging of RCC. The primary treatment for localized RCC is surgery, whereas, immunotherapy, targeted drugs, and chemotherapy are preferred treatments for advanced and metastatic RCC [3]. Although the diagnostic and therapeutic methods have improved significantly, the incidence and mortality rates of ccRCC are high and are increasing [1]. Therefore, novel biomarkers are necessary for early detection of ccRCC in order to reduce mortality rates.

Autophagy is an important biological process that maintains cellular homeostasis by degrading aged or damaged proteins and organelles within the lysosomes [4, 5]. Autophagy plays dual roles in tumorigenesis and non-neoplastic diseases [6, 7]. Higher levels of autophagy in cardiomyocytes are associated with heart failure [8, 9]. Conversely, in ischemic heart disease, induction of autophagy is required to maintain energy homeostasis and survival of cardiomyocytes [10]. In early stages of many cancers, autophagy suppresses the transformation and growth of cancer cells. However, in later stages of tumors, autophagy promotes rapid growth of malignant cells by degrading and recycling components of damaged or aged organelles to meet their metabolic demands for rapid growth [11]. Therefore, the levels of autophagy proteins can regulate tumor growth and progression [12].

Previous studies have shown that autophagy plays a vital role in the growth and progression of ccRCC. Hall et al. showed that TRPM3 expression promotes the growth of VHL*-*negative ccRCC cells by enhancing autophagy under starvation conditions [13]. Mikhaylova et al. reported that LC3B-mediated autophagy promotes the growth of VHL-negative ccRCC tumors [14]. The role of autophagy in the progression of ccRCC has been shown in several studies by following the functional status of one or more autophagy proteins [15, 16]. However, the role of the entire subset of autophagy genes in the prognosis of ccRCC has not been studied.

In the present study, we explored the prognostic significance of autophagy-related genes (ARGs) in ccRCC tumors using information derived from high-throughput expression profiles in public databases. We identified 38 differentially expressed autophagy-related genes (DEARGs) in ccRCC tissues. Furthermore, a combination of Lasso regression and Cox regression analyses showed that expression of five DEARGs was associated with overall survival (OS) and expression of four DEARGs correlated with disease-free survival (DFS) in ccRCC patients. We constructed two Cox regression models (OS model and DFS model) using these DEARGs and assessed the specificity and sensitivity of these models to determine prognostic significance using ROC curve analysis. Our data suggests that both the models accurately predict patient prognosis. Finally, we analyzed the diagnostic value of the DEARGs by assessing the relationship between the expression of the DEARGs and the drug sensitivities of the ccRCC cell lines. Our data showed a high area under the curve (AUC) value for all the DEARGs we analyzed, thereby demonstrating their potential significance in diagnosing ccRCC. We further identified *BIRC5* as a promising therapeutic target for ccRCC.

## RESULTS

### Identification of differentially expressed ARGs in ccRCC tissue samples

**Figure 1:**
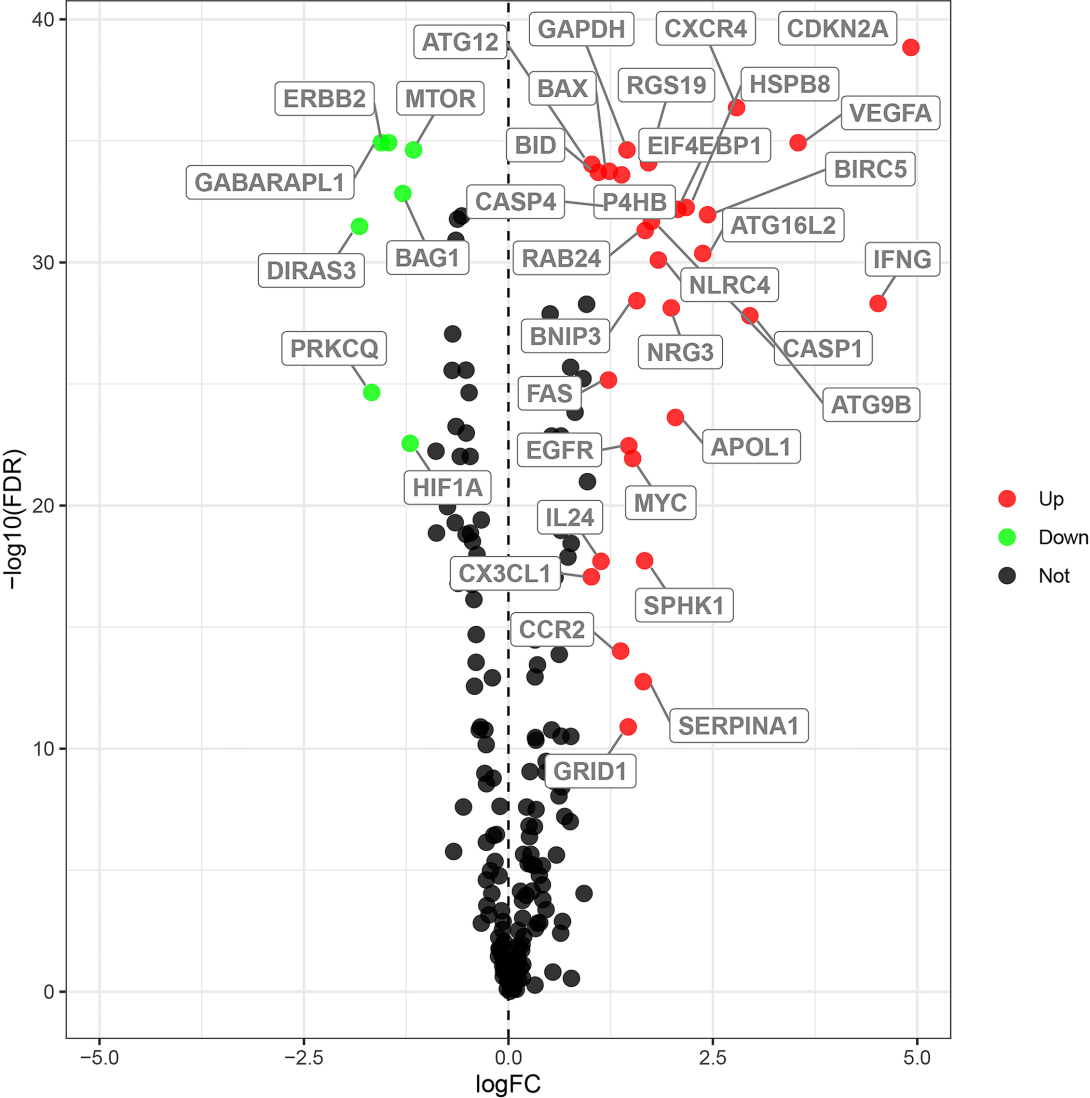
**Differential expression of autophagy-related genes in ccRCC tissue samples.** The differential expression of 238 autophagy related genes (ARGs) in ccRCC tissue samples (n=539) compared with normal healthy kidney samples (n=72) is shown in the –log (FDR) vs. log (FC) plot. The red dots represent 31 upregulated DEARGs, the green dots represent 7 downregulated DEARGs, and the remaining black dots represent ARGs that are not differentially expressed in ccRCC tissue samples.

We analyzed the expression of 232 ARGs in 539 ccRCC and 72 normal kidney tissue samples using the Wilcoxon signed-rank test and identified 38 differentially expressed ARGs (DEARGs). This included 31 upregulated DEARGs and 7 downregulated DEARGs (FDR < 0.05, |log2FC| > 1; Figure 1).

### Identification of prognostic risk DEARGs in the training group ccRCC patients

Next, we performed univariate Cox regression analysis of the expression of the 38 DEARGs in the training group ccRCC patients to identify prognostic DEARGs. The data showed that expression of 19 DEARGs each significantly correlated with the OS (*p* < 0.05) and the DFS (*p* < 0.05) of ccRCC patients (Supplementary Figure 1). We performed Lasso regression analysis to eliminate false-positive DEARGs. Subsequently, our analysis showed that the expression of 12 DEARGs correlated with the OS of ccRCC patients, and the expression of 6 DEARGs correlated with the DFS of ccRCC patients (Supplementary Figure 2). We used these 18 DEARGs (12 for OS and 6 for DFS) to construct models for predicting the prognosis of ccRCC patients.

**Figure 2:**
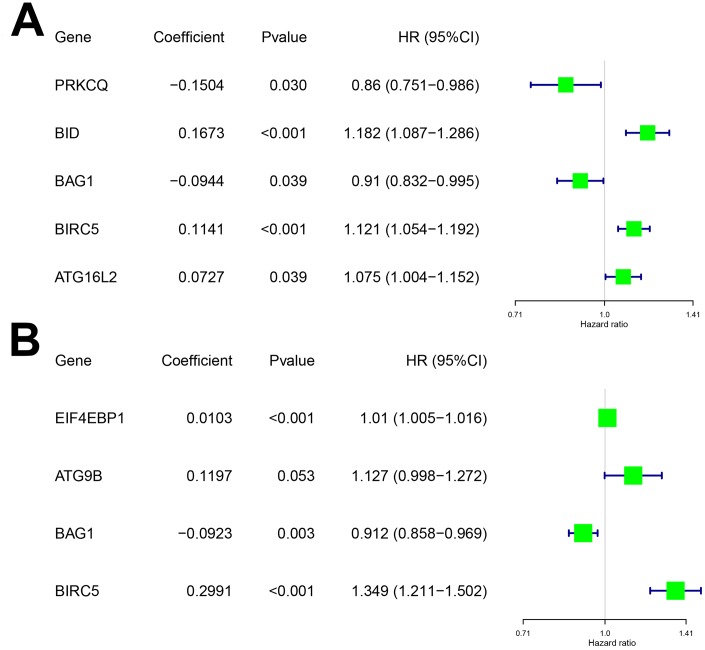
**Characteristics of risk DEARGs in the prognostic risk models.** Regression coefficients and hazard ratios of the risk DEARGs for the (**A**) Overall survival (OS) and (**B**) Disease-Free Survival (DFS) models are shown.

To determine the optimal model for predicting prognosis, we performed multivariate Cox proportional hazards regression analysis using forward and backward selection algorithms. We identified *PRKCQ*, *BID*, *BAG1*, *BIRC5*, and *ATG16L2* as risk genes in the OS model and *EIF4EBP1*, *BAG1*, *ATG9B*, and *BIRC5* as risk genes in the DFS model. The high-risk DEARGs negatively correlated with patient prognosis, whereas the low-risk DEARGs positively correlated with patient prognosis. We identified *BID*, *BIRC5*, and *ATG16L2* as high risk genes and *BAG1* and *PRKCQ* as low risk genes in the OS model; similarly, we identified *EIF4EBP1*, *ATG9B*, and *BIRC5* as high risk and *BAG1* as a low risk gene in the DFS model (Figure 2A–2B and Table 1).

**Table 1:** List of the risk DEARGs associated with ccRCC patient prognosis.

**Gene name**	**Gene ID**	**Protein name**	**Location**	**Expression status**
*PRKCQ*	5588	Protein kinase C theta	Chromosome 10	Downregulated
*BID*	637	BH3 interacting domain death agonist	Chromosome 22	Upregulated
*BAG1*	573	BCL2 associated athanogene 1	Chromosome 9	Downregulated
*BIRC5*	332	Baculoviral IAP repeat containing 5	Chromosome 17	Upregulated
*ATG16L2*	89849	Autophagy related 16 like 2	Chromosome 11	Upregulated
*EIF4EBP1*	1978	Eukaryotic translation initiation factor 4E binding protein 1	Chromosome 8	Upregulated
*ATG9B*	285973	Autophagy related 9B	Chromosome 7	Upregulated

### Testing the prognostic risk models in the training group

We used the following formula to calculate the prognostic risk scores for the training groups using the gene expression values and the regression coefficients of the risk genes: Training group risk score for OS = (-0.1504 × expression value of *PRKCQ*) + (0.1673 × expression value of *BID*) + (-0.0944 × expression value of *BAG1*) + (0.1141 × expression value of *BIRC5*) + (0.0727 × expression value of *ATG16L2*); Training group risk score for DFS= (0.0103 × expression value of *EIF4EBP1*) + (0.1197 × expression value of *ATG9B*) + (-0.0923 × expression value of *BAG1*) + (0.2991 × expression value of *BIRC5*).

**Figure 3:**
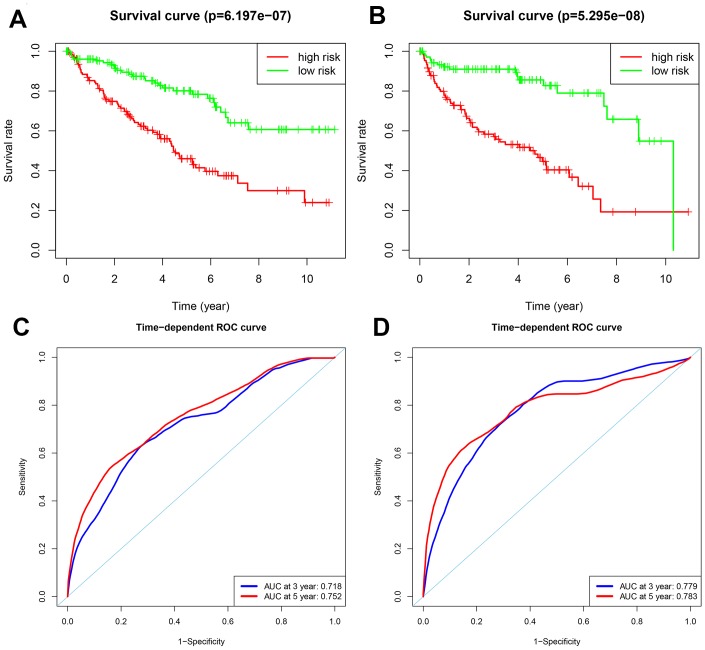
**Analysis of OS and DFS prognostic risk models in training group ccRCC patients.** (**A**) Kaplan-Meier survival curve analysis of OS in the high-risk (red line) and low-risk (green line) ccRCC patients in the training group. (**B**) Kaplan-Meier survival curve analysis of disease-free survival (DFS) in the high-risk (red line) and low-risk (green line) ccRCC patients. (**C**) Time-dependent ROC curves show area under curve (AUC) values at 3-year (blue) and 5-year (red) OS in the training group ccRCC patients. (**D**) Time-dependent ROC curves show AUC values at 3-year (blue) and 5-year (red) DFS in the training group ccRCC patients.

Patients in the training group were subdivided into high and low risk groups for OS (n = 133 each) and DFS (n=108 each) based on the median risk scores. Kaplan-Meier survival curve analysis using the log-rank test showed that the high-risk groups exhibited worse prognosis than the low-risk groups (Figure 3A–3B; all *p* < 0.05). The 3-year and 5-year OS rates for the high-risk patients were 63.3% and 46.0%, respectively, whereas, the 3-year and 5-year OS rates for the low-risk patients were 87.4% and 80.1%, respectively. The 3-year and 5-year DFS rates for the high-risk patients were 58.3.7% and 44.6.8%, respectively. The 3-year and 5-year DFS rates for the low-risk training group patients were 91.0% and 85.6%, respectively.

**Figure 4:**
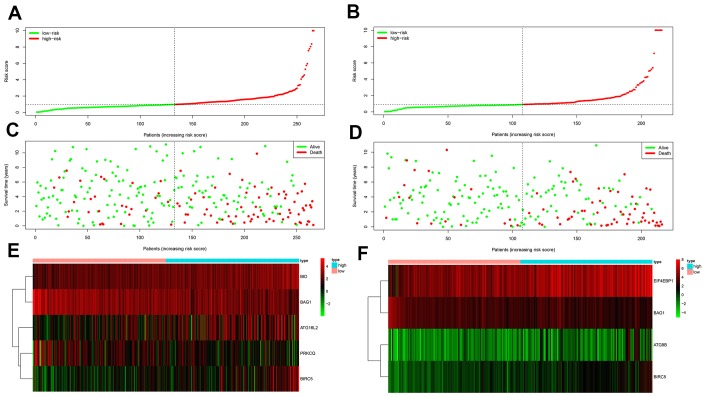
**Prognosis of high-risk and low-risk training group ccRCC patients.** (**A**) Risk score distribution of high-risk (red) and low-risk (green) ccRCC patients in the OS model. (**B**) Risk score distribution of high-risk (red) and low-risk (green) ccRCC patients in the DFS model. (**C**) Scatter plot shows the survival status of ccRCC patients in the OS model. Red dots denote patients that are dead and green dots denote patients that are alive. (**D**) Scatter plot shows survival status of ccRCC patients in the DFS model. Red dots denote patients that are dead and green dots denote patients that are alive. (**E**) Expression of risk genes in the high-risk (blue) and low-risk (pink) training group ccRCC patients in the OS model. (**F**) Expression of risk genes in the high-risk (blue) and low-risk (green) training group ccRCC patients in the DFS model. The color code for gene expression in E and F shows green denoting lowest expression and red denoting highest expression

We then measured the predictive performance of the prognostic risk models for the 3-year and 5-year OS and DFS using the time-dependent receiver operating characteristic (ROC) curves. The area under the ROC (AUC) values for the two prognostic models were 0.718 (OS) and 0.779 (DFS) at 3 years, and 0.752 (OS) and 0.783 (DFS) at 5 years (Figure 3C–3D). We then ranked the risk scores of patients for OS and DFS and analyzed their distribution (Figure 4A–4B). The dot plots show the OS and DFS status of individual ccRCC patients in the training groups (Figure 4C–4D). The heat maps show the expression patterns of the risk genes in the high- and low-risk patient groups (Figure 4E–4F). As shown, patients with high-risk scores in the OS group showed upregulation of *BID*, *BIRC5*, and *ATG16L2* (high risk genes) and downregulation of *PRKCQ* and *BAG1* (protective gene), whereas, patients with low-risk scores showed downregulation of *BID*, *BIRC5*, and *ATG16L2* and upregulation of *PRKCQ* and *BAG1*. Moreover, patients with high-risk groups in the DFS group showed upregulation of *EIF4EBP1*, *ATG9B*, and *BIRC5* (high risk genes) and downregulation of *BAG1* (protective gene), whereas, patients with low-risk groups showed downregulation of *EIF4EBP1*, *ATG9B*, and *BIRC5* and upregulation of the *BAG1*.

### Verification of the prognostic models in the testing group

**Figure 5:**
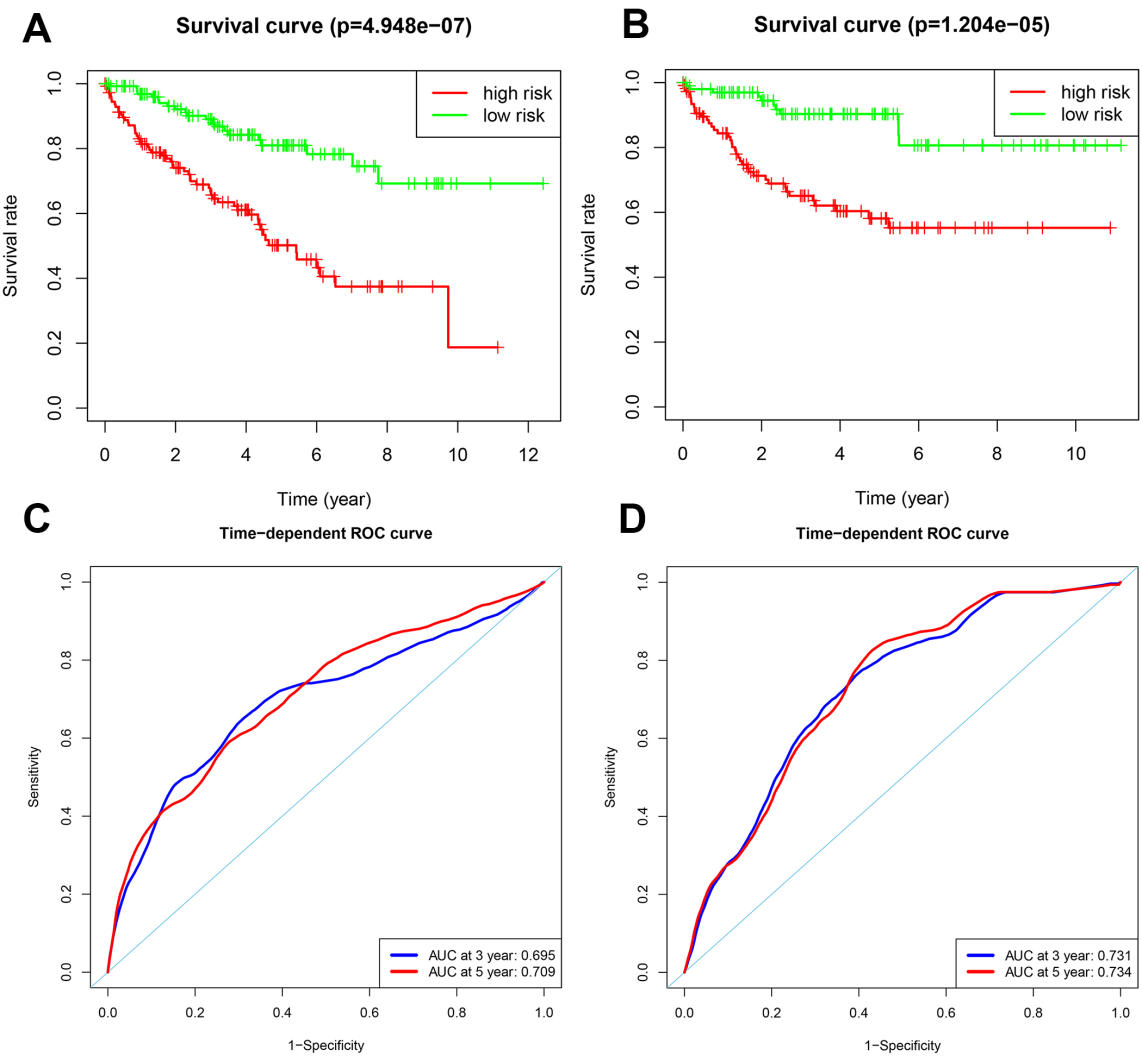
**Validation of the OS and DFS prognostic risk models in the testing group ccRCC patients.** (**A**) Kaplan-Meier survival curve analysis of OS in the high-risk (red line) and low-risk (green line) ccRCC patients in the testing group. (**B**) Kaplan-Meier survival curve analysis of DFS in the high-risk (red line) and low-risk (green line) ccRCC patients in the testing group. (**C**) Time-dependent ROC curve analyses shows AUC values for 3-year (blue) and 5-year (red) OS in the testing group ccRCC patients. (**D**) Time-dependent ROC curve analyses shows AUC values for 3-year (blue) and 5-year (red) DFS in the testing group ccRCC patients.

**Figure 6:**
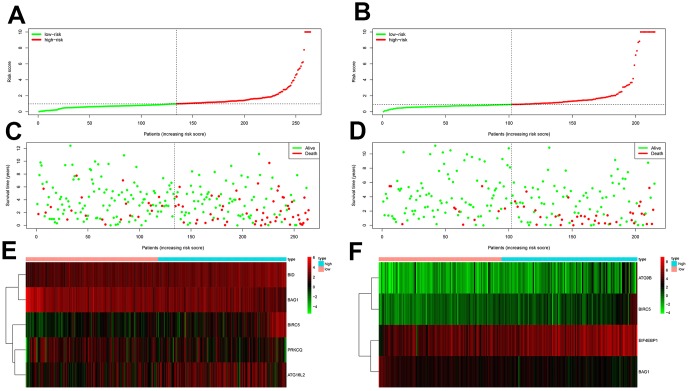
**Prognostic analyses of high-risk and low-risk ccRCC patients in the testing group.** (**A**) Risk score distribution of high risk (red) and low-risk (green) ccRCC patients from the testing group using the OS model. (**B**) Risk score distribution of high risk (red) and low-risk (green) ccRCC patients from the testing group using the DFS model. (**C**) Scatter plots show survival status of testing group ccRCC patients using the OS model. (**D**) Scatter plots show survival status plots of testing group ccRCC patients using the DFS model. (**E**) Expression of risk genes in the high-risk (blue) and low-risk (pink) testing group ccRCC patients in the OS model. (**F**) Expression of risk genes in the high-risk (blue) and low-risk (pink) testing group ccRCC patients in the DFS model. The color code for gene expression in E and F shows green denoting lowest expression and red denoting highest expression.

We validated the accuracy of the two prognostic risk models by analyzing the patients in the testing group. We calculated the risk scores of each patient based on the expression of the risk genes for the OS model, namely, *PRKCQ*, *BID*, *BAG1*, *BIRC5*, and *ATG16L2*, and the risk genes for the DFS model, namely, *EIF4EBP1*, *BAG1*, *ATG9B*, and *BIRC5*. We subdivided the testing group patients into high-risk (n = 130) and low-risk (n = 134) groups in the OS model, and high-risk (n = 113) and low-risk (n = 102) groups in the DFS model. Kaplan-Meier survival curve analysis showed significant differences between the high- and the low-risk groups for OS (*p* < 0.05) and DFS (*p* < 0.05), respectively (Figure 5A–5B). Our analysis showed that the 3-year and 5-year OS rates for the high-risk group were 66.8% and 50.2%, respectively, and 89.0% and 81.0%, respectively, for the low-risk groups. The 3-year and 5-year DFS rates for the high-risk group were 65.1% and 58.1%, respectively, and 90.3% and 87.1%, respectively, for the low-risk group. We further performed ROC curve analyses of OS and DFS at 3 years and 5 years for the testing patients based on the two prognostic risk models. The AUC values for the OS model at 3 years and 5 years were 0.695 and 0.709, respectively, whereas, the AUC values for the DFS model at 3 years and 5 years were 0.731 and 0.734, respectively (Figure 5C–5D). The distribution of risk score, survival status, and the expression of risk genes in the testing group patients are shown in Figure 6A–6F.

Our data showed that for both OS and DFS, the protective genes were upregulated and the risk genes were downregulated in the low-risk groups, whereas, the protective genes were downregulated and the risk genes were upregulated in the high-risk groups. These results confirmed that both the risk models accurately predicted the prognosis of ccRCC patients

### Both prognostic risk models are independently associated with OS and DFS of ccRCC patients from the TCGA group

We further analyzed the relationship between the risk scores of the two models and clinical parameters such as age, gender, histological grade, and the pathological. stage. As shown in Table 2, univariate Cox regression analysis showed that age, histological grade, pathological stage, and the risk score correlated with OS of ccRCC patients (p < 0.05). Similarly, histological grade, pathological stage, and risk score associated with DFS of ccRCC patients (p < 0.05). Next, we performed multivariate Cox regression analysis using age, gender, histological grade, pathological stage, and the risk scores obtained from the prognostic models as explanatory variables. As shown in Table 2, both the prognostic models independently correlated with OS and DFS of all patients from the TCGA group (p < 0.05). Furthermore, multivariate analysis showed that histological grade and pathological stage significantly correlated with OS and DFS (p < 0.05). Moreover, age of the patients significantly correlated with OS (p < 0.05). These results demonstrate that both prognostic models can be independently used to predict OS and DFS in ccRCC patients.

**Table 2:** Univariate and multivariate cox regression analyses of OS and DFS in the TCGA group ccRCC patients.

**Variables**	**Univariate analysis**		**Multivariate analysis**	
	**HR (95% CI)**	***p*-Value**	**HR (95% CI)**	***p*-Value**
**Overall survival (OS)**				
Risk model (high/low)	1.10 (1.07-1.13)	2.13E-11	1.06 (1.03-1.10)	5.68E-06
Age	1.02 (1.01-1.04)	2.16E-05	1.02 (1.01-1.04)	6.03E-05
Gender	0.96 (0.70-1.31)	0.797		
Histological grade	2.27 (1.85-2.78)	3.00E-15	1.47 (1.17-1.85)	0.000
Pathological stage	1.87 (1.64-2.13)	1.10E-20	1.65 (1.42-1.91)	6.06E-11
**Disease free survival (DFS)**				
Risk model (high/low)	1.43 (1.23-1.66)	1.41E-06	1.28 (1.01-1.62)	0.033
Age	1.00 (0.99-1.02)	0.251		
Gender	1.46 (0.97-2.18)	0.062		
Histological grade	2.96 (2.28-3.82)	1.38E-16	1.68 (1.28-2.20)	0.000
Pathological stage	2.63 (2.21-3.14)	2.78E-27	2.33 (1.93-2.81)	9.84E-19

**Figure 7:**
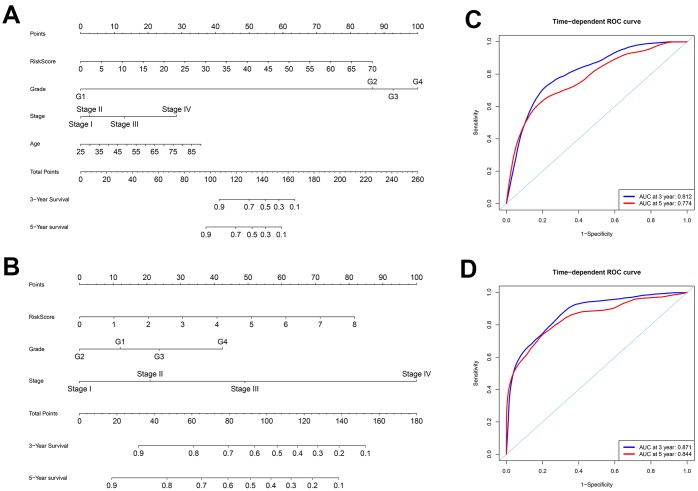
**Construction of nomograms and ROC curve analysis of prognosis for ccRCC patients from the TCGA database.** (**A**–**B**) The nomograms for (**A**) OS and (**B**) DFS are shown. (**C**–**D**) ROC curve analysis shows 3-year (blue) and 5-year (red) OS and the corresponding AUC values for the ccRCC patients from the TCGA database. (**D**) ROC curve analysis shows 3-year (blue) and 5-year (red) DFS and the corresponding AUC values for the ccRCC patients from the TCGA database.

To better predict prognosis at 3- and 5-years post-surgery for ccRCC patients, we constructed two new nomograms using variables associated with OS (age, histological grade, pathological stage, and risk score) and DFS (histological grade, pathological stage, and risk score), respectively (Figure 7A–7B). We then assessed the accuracy of the nomograms using the ROC curve analysis to determine the area under ROC curve (AUC) values. The AUC values for 3-year OS and DFS were 0.812 and 0.871, respectively, whereas the AUC values for 5-year OS and DFS were 0.774 and 0.844, respectively (Figure 7C–7D). These data suggested that both nomograms accurately predicted the 3-year and 5-year OS and DFS rates after surgery in the ccRCC patients.

### Diagnostic value of risk DEARGs in ccRCC

**Figure 8:**
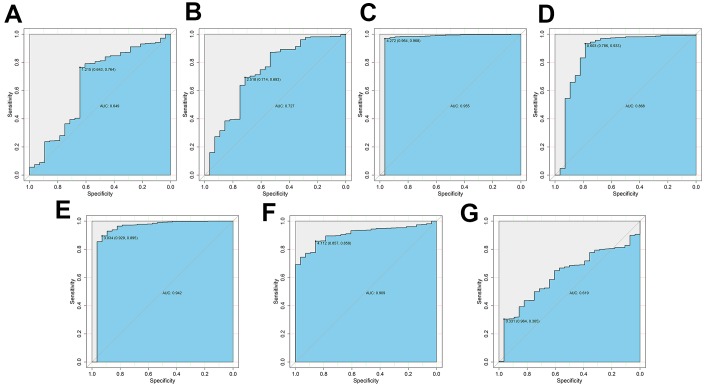
**ROC curve analysis to determine potential diagnostic value of the risk DEARGs in ccRCC.** The ROC curve plots for (**A**) *PRKCQ* (AUC = 0.649), (**B**) *BID* (AUC = 0.727), (**C**) *BAG1* (AUC = 0.955), (**D**) *BIRC5* (AUC = 0.868), (**E**) *ATG16L2* (AUC = 0.942), (**F**) *EIF4EBP1* (AUC = 0.909), and (**G**) *ATG9B* (AUC = 0.619) genes in ccRCC are shown.

We then assessed the diagnostic values of the risk genes for OS (*PRKCQ*, *BID*, *BAG1*, *BIRC5*, and *ATG16L2*) and DFS (*EIF4EBP1*, *BAG1*, *ATG9B*, and *BIRC5*) using ROC curve analysis of gene expression data from 535 ccRCC patients from the TCGA database and 28 healthy individuals from the Genotype-Tissue Expression project. The AUC values for *PRKCQ,* *BID, BAG1,* *BIRC5, ATG16L2, EIF4EBP,* and *ATG9B* genes were 0.649, 0.727, 0.955, 0.868, 0.942, 0.909 and 0.619, respectively (Figure 8). This demonstrated that risk DEARGs are potential diagnostic markers.

### Drug sensitivities of ccRCC cell lines

**Figure 9:**
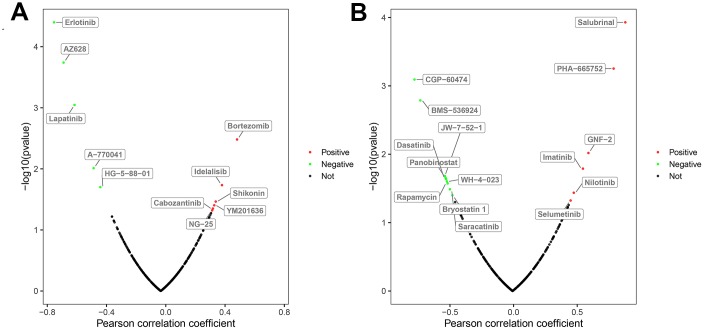
**Correlation between the expression status of risk DEARGs and drug sensitivity of ccRCC cell lines.** The plot shows the correlation between the expression status of (**A**) *BAG1* and (**B**) *BIRC5* genes relative to the sensitivity of several ccRCC cell lines to various drugs. The green dots represent drugs that negatively correlate with the expression of the risk genes (*p* < 0.05) based on their IC50 values; red dots indicate positive correlation of the corresponding drugs with the expression of risk genes (*p* < 0.05) based on their IC50 values; and black dots represent drugs that do not show any significant correlation based on their IC50 values with the expression of risk genes (*p* > 0.05).

Next, we used the GDSC database to analyze the relationship between the drug sensitivity of several ccRCC cell lines and the expression of *BAG1* and *BIRC5*, which are risk DEARGs for OS and DFS, respectively. Initially, we analyzed the relationship between the expression of these two genes and the IC50 (natural log half maximal inhibitory concentration) values of several targeted drugs in the ccRCC cell lines. We hypothesized that positive correlation between the risk DEARG expression and the IC50 value would imply increased drug resistance in the ccRCC cell lines. Conversely, negative correlation between the risk DEARG expression and IC50 value would suggest increased drug sensitivity in the ccRCC cell lines. Our results indicated that high expression of *BAG1* increased the resistance of ccRCC cell lines to drugs such as Bortezomib, Idelalisib, Shikonin, YM201636, Cabozantinib, and NG-25 (*p* < 0.05), and increased sensitivity of ccRCC cell lines to other drugs such as Erlotinib, AZ628, Lapatinib, A-770041, and HG-5-88-01(*p* < 0.05; Figure 9A). On the other hand, high expression of *BIRC5* expression increased resistance of ccRCC cell lines to drugs such as Salubrinal, PHA-665752, GNF-2, Imatinib, Nilotinib, and Selumetinib (*p* < 0.05), and increased sensitivity of ccRCC cell lines to other drugs such as CGP-60474, BMS-536924, JW-7-52-1, Panobinostat, Dasatinib, WH-4-023, Rapamycin, Saracatinib, and Bryostatin 1(*p* < 0.05; Figure 9B).

**Figure 10:**
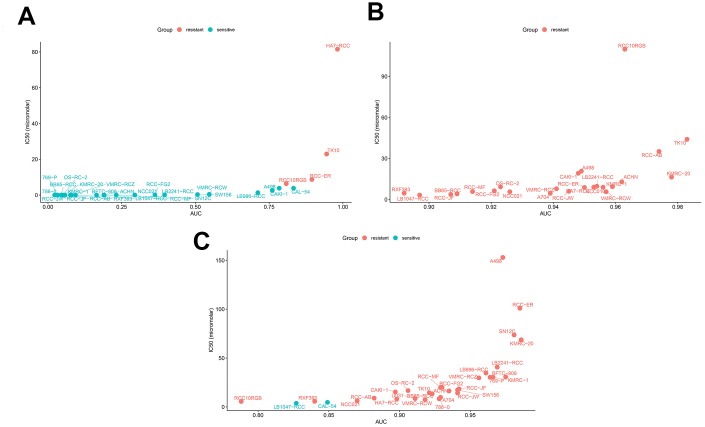
**Drug sensitivity analyses of ccRCC cell lines.** The AUC versus IC50 plots show sensitivity of several ccRCC cell lines to treatment with (**A**) Sepantronium bromide, (**B**) Axitinib, and (**C**) Cabozantinib. The cell lines with IC50 values that are greater than the maximum screening concentrations used for the targeted drugs are considered to be resistant to the corresponding drugs. The green dots denote drug-sensitive ccRCC cell lines and red dots denote drug-resistant ccRCC cell lines. IC50 =half maximal inhibitory concentration; AUC: Area under the dose-response curve.

We also compared the sensitivity of ccRCC cell lines to a *BIRC5*-targeted drug (Sepantronium bromide) against conventional targeted drugs (Axitinib and Cabozantinib). Our data suggested that the sensitivity of ccRCC cell lines to the *BIRC5*-targeted drug, Sepantronium bromide, was significantly higher than the sensitivity to conventional targeted drugs (Figure 10A–10C; Table 3; *p* < 0.05). These results indicate that *BIRC5* is a potential therapeutic target for ccRCC.

**Table 3:** Drug sensitivity analysis of ccRCC cell lines.

**Sepantronium bromide vs. Axitinib**						**Sepantronium bromide vs. Cabozantinib**					
**Drug**	**Total (N)**	**Sensitive**	**Resistant**	**χ^2^**	***p*-Value**	**Drug**	**Total (N)**	**Sensitive**	**Resistant**	**χ^2^**	***p*-Value**
Sepantronium bromide	29	25	4	35.78	2.209E-09	Sepantronium bromide	29	25	4	35.353	2.75E-09
Axitinib	24	0	24			Cabozantinib	31	2	29		

## DISCUSSION

Previous studies have reported that ccRCC is a malignant disease that involves reprogramming of mechanisms involved in energetic metabolism, such as aerobic glycolysis, over-utilization of amino acids like tryptophan, glutamine, and arginine, tricarboxylic acid (TCA) cycle, and dysfunctional mitochondrial bioenergetics and oxidative phosphorylation [17, 18]. Several studies have previously shown that inhibition or upregulation of autophagy modulates metabolic reprogramming of cancer cells [19, 20]. Currently, the role of autophagy in ccRCC is controversial. Studies suggest that autophagy suppresses cancer initiation, but, promotes cancer progression and modulates cancer responses to various therapies [11, 21]. Therefore, investigating the expression patterns of ARGs is essential for understanding the role of autophagy in ccRCC. Although the correlation between individual ARG**s** and ccRCC has been explored in previous studies [13, 14, 22], in-depth analysis of all the ARGs has not been analyzed. Furthermore, the correlation between the expression of ARGs and prognosis of ccRCC patients has not been established.

In the present study, we analyzed the high-throughput RNA-seq data from the TCGA database to identify key ARGs that are relevant for diagnosis, treatment, and prognosis predictions in ccRCC patients. We found that 38 out of 232 ARGs were differentially expressed in ccRCC patient tumor samples, including 31 up-regulated and 7 down-regulated genes. Then, we used Cox regression and Lasso regression analyses and identified 5 risk DEARGs (*PRKCQ*, *BID*, *BAG1*, *BIRC5*, and *ATG16L2*) that were associated with OS and 4 risk DEARGs (*EIF4EBP1*, *BAG1*, *ATG9B*, and *BIRC5*) that correlated with DFS. We further constructed two prognostic risk models (OS model and DFS model) using the risk DEARGs and demonstrated that they could provide an accurate prognosis of ccRCC patients. Multivariate Cox regression analysis of the two prognostic models and other clinical parameters suggested that the expression of risk DEARGs independently predicted the prognosis of ccRCC patients. Furthermore, ROC curve analysis showed that the expression of the risk DEARGs accurately distinguished healthy individuals from ccRCC patients suggesting diagnostic significance. Finally, we analyzed the relationship between the expression *BIRC5* and *BAG1* genes and drug sensitivity in ccRCC cell lines. Our results suggest that level of *BIRC5* and *BAG1* expression correlates with drug sensitivity in ccRCC cell lines. Several ccRCC cell lines were sensitive to the *BIRC5*-targeted drug, Sepantronium bromide, thereby suggesting that *BIRC5* is a potential therapeutic target for ccRCC patients.

Previous studies have shown that over-expression of *PRKCQ*, a member of the novel PKC family significantly enhances growth, migration and drug resistance of breast cancer cells; furthermore, silencing of *PRKCQ* inhibits breast cancer cell growth by promoting apoptosis [23]. *PRKCQ* promotes breast cancer progression by enhancing the transcriptional activity via phosphorylation of the AP-1 transcription factor, Fra-1 [24]. In contrast, *PRKCQ* is significantly down-regulated in ccRCC and chromophobe renal cell carcinomas (ChRCCs), [25, 26]. Our study found that *PRKCQ* was down-regulated in ccRCC tissues and its down-regulation correlated with worse prognosis in the ccRCC patients.

*BID* is found on chromosome 22q11.21 and encodes an apoptosis-related protein that heterodimerizes with BAX, an activator of apoptosis, or BCL2, a negative regulator of apoptosis. *BID* is over-expressed in thyroid cancer and associated with patient prognosis [27]. The role of *BID* in ccRCC is unclear. In this study, we found that overexpression of *BID* is associated with poor prognosis of ccRCC patients.

*BAG1* is located on chromosome 9 in humans and encodes a membrane protein that regulates apoptosis or programmed cell death. In gastric cancer, over-expression of *BAG1* is associated with tumor progression and its silencing promotes gastric cancer cell apoptosis [28]. *BAG1* expression is up-regulated in acute myeloid leukemia (AML), and its silencing promotes AML cell apoptosis [29]. In RCC, downregulation of *BAG1* correlates with poor patient prognosis [30]. In this study, we found that upregulation of *BAG1* in ccRCC correlates with poor patient prognosis.

*ATG16L2* is located on chromosome 11 and has not been studied in relation to cancer. In this study, we found that high expression of *ATG16L2* in ccRCC tissues associated with poor patient prognosis The *EIF4EBP1* gene is located at Ch.8p11.23 and encodes a translation repressor protein. High expression of *EIF4EBP1* in hepatocellular carcinoma tissues correlates with poor patient prognosis [31]. Rutkovsky et al. showed that over-expression of *EIF4EBP1* in breast cancer was associated with poor patient prognosis, and it’s silencing significantly inhibited breast cancer cell proliferation by promoting G1 cell cycle arrest [32]. In this study, we found that high expression of *EIF4EBP1* was associated with worse patient prognosis.

*ATG9B* is located on chromosome 7 in humans, and is involved in the regulation of autophagy. Wang et al. reported that *ATG9B* expression was downregulated in hepatocellular carcinoma and suppression of ATG9B expression in hepatocytes promoted tumorigenesis [33]. A previous study showed that upregulation of ATG9B expression correlated with ccRCC progression [34]. Our study demonstrates that overexpression of *ATG9B* in ccRCC tissues is associated with worse patient prognosis.

High *BIRC5* expression is associated with tumor progression in lung adenocarcinoma and poor patient prognosis [35]. In acute myelocytic leukemia (AML), knock-down of *BIRC5* induces apoptosis and is therefore regarded as a potential therapeutic target for AML patients [36]. In this study, we demonstrate that high *BIRC5* expression is associated with worse prognosis in ccRCC patients and elevated drug resistance in ccRCC cell lines. Therefore, we postulate that *BIRC5* is a potential therapeutic target for ccRCC patients.

The main limitation of our findings is that our study was conducted using data already available from patients in several public databases. These findings need to be validated in prospective clinical trials. Moreover, the mechanisms through which ARGs modulate the initiation and progression of ccRCC requires further investigation.

In summary, our study demonstrates that differentially expressed ARGs have great potential as diagnostic and prognostic biomarkers and therapeutic targets in ccRCC. Further investigations are necessary to confirm the findings of our study.

## MATERIALS AND METHODS

### Patient information and databases

We obtained a list of 232 human autophagy-related genes from the Human Autophagy Database (HADb; http://autophagy.lu/clustering/index.html). The gene expression profiles (HTSeq - FPKM) of 611 patients with ccRCC were downloaded from the TCGA database (https://portal.gdc.cancer.gov/), and the patient clinical data was obtained from the cBioPortal database (https://www.cbioportal.org/). The data was analyzed using the R software (https://www.r-project.org/). First, we matched the ID numbers of the patients with their corresponding gene expression profile and clinical data and excluded patients whose ID numbers did not match. We obtained complete gene expression profiles and overall survival (OS) data from 530 patients and complete gene expression profiles and disease-free survival (DFS) data from 431 patients (Table 4). We used the UCSC database (https://xena.ucsc.edu/) to download gene expression profiles (RNA-seq) from the normal kidney tissues of healthy individuals from the Genotype-Tissue Expression project (n=28) and ccRCC tissues from the TCGA database (n = 535). To ensure a unified standard, the RNA-seq profiles were transformed using the formula log_2_(x+1) and normalized. The drug sensitivity data for the ccRCC cell lines was downloaded from the Genomics of Drug Sensitivity in Cancer (GDSC) database (https://www.cancerrxgene.org/).

**Table 4:** Clinical data of 530 ccRCC patients.

**Clinical parameters**	**Variable**	**Total (530)**	**Percentages (%)**
Age	<60	245	46.0%
	≥60	285	54.0%
Gender	Female	186	35.1%
	Male	344	64.9%
Histological grade	G1	14	2.6%
	G2	227	42.8%
	G3	206	38.9%
	G4	75	14.2%
	GX	5	1.0%
	Unknown	3	0.5%
Pathological stage	Stage I	265	50.0%
	Stage II	57	10.8%
	Stage III	123	23.2%
	Stage IV	82	15.5%
	Unknown	3	0.5%
Disease-free Status	Recurred/Progressed	123	23.2%
	Disease free	308	58.1%
	Unknown	99	18.7%
Survival status	Dead	173	32.6%
	Alive	357	67.4%

### Identification of DEARGs

We identified the differentially expressed ARGs (DEARGs) using the Wilcoxon signed-rank test. The cut-off values were determined according to the parameters, false discovery rate (FDR) < 0.05 and |log_2_ fold change (FC)| > 1.

### Construction of OS and DFS risk prognostic models

We randomly divided the 530 patients with complete OS information into two groups, namely, a training group of OS (n = 266) and a testing group of OS (n = 264; Table 5). We also randomly divided the 431 patients with complete DFS information into two groups, namely, a training group of DFS (n = 216) and a testing group of DFS (n = 215; Table 5). We constructed the Cox regression models for OS and DFS using the data from the training group and validated its accuracy using the testing group. Initially, the potential prognostic genes were selected using the univariate Cox regression analysis. Lasso regression analysis was used to eliminate false positives because of over-fitting. Finally, Cox proportional hazards regression was used to construct the OS and DFS prognostic risk models.

**Table 5:** Survival and disease-free status of ccRCC patients from different groups.

**Clinical parameters**	**Variables**	**Training group**	**Testing group**	**TCGA group**
Survival status	Dead	96 (18.1%)	77 (14.5%)	173 (32.6%)
	Alive	170 (32.1%)	187 (35.3%)	357 (67.4%)
Disease free Status	Recurred/Progressed	74 (17.2%)	49 (11.3%)	123 (28.5%)
	Disease-free	142 (33.0%)	166 (38.5%)	308 (71.5%)

### Risk score calculation

The risk score for each patient was calculated using the regression coefficients of the individual genes obtained from the multivariate Cox regression model and the expression value of each of the selected genes. The computational formula used for this analysis was

$\text{Risk}\;\text{score}\;(\text{patient})=\sum_{i=1,2,...,n}\text{coefficient}\;(\text{genei})\times \text{expression}$

where gene_*i*_ represents the identifier of the _*i*_th selected genes and coefficient (gene_*i*_) value is the estimated regression coefficient of the gene_*i*_ derived from the Cox proportional hazards regression analysis. The risk score is a measure of prognostic risk for each ccRCC patient. We classified the ccRCC patients into high-risk and low-risk groups using the median risk score of the training group as the cutoff. A high-risk score indicates poor prognosis for the ccRCC patients.

### Statistical analysis

All statistical analyses were performed using the R software, and *p* < 0.05 was regarded as statistically significant. The distribution differences among the variables were assessed using the chi-square test or the Fisher’s exact test. The Kaplan-Meier survival curve analysis and the log-rank test were used to analyze OS and DFS. The Cox regression model was used to analyze the factors that affect the survival of ccRCC patients. Univariate and multivariate analyses were performed using the Cox proportional hazards regression model. Time-dependent ROC analysis was used to evaluate the accuracy of the models that predicted prognosis. ROC curve analysis was also used to estimate the diagnostic value of gene expression. An area under the curve (AUC) value of 0.75 or greater was considered a significant predictive value, and values equal to or greater than 0.6 were regarded as acceptable for predictions.

## Supplementary Material

Supplementary Figures
