# Supplementary material for: Differentially expressed autophagy-related genes are potential prognostic and diagnostic biomarkers in clear-cell renal cell carcinoma
Source: Aging (Albany NY). 2019 Oct 17;11(20):9025–42. doi: 10.18632/aging.102368 (PMC6834403; doi:10.18632/aging.102368)
Supplement: Supplementary Figures [file aging-11-102368-s001.pdf]

SUPPLEMENTARY FIGURES

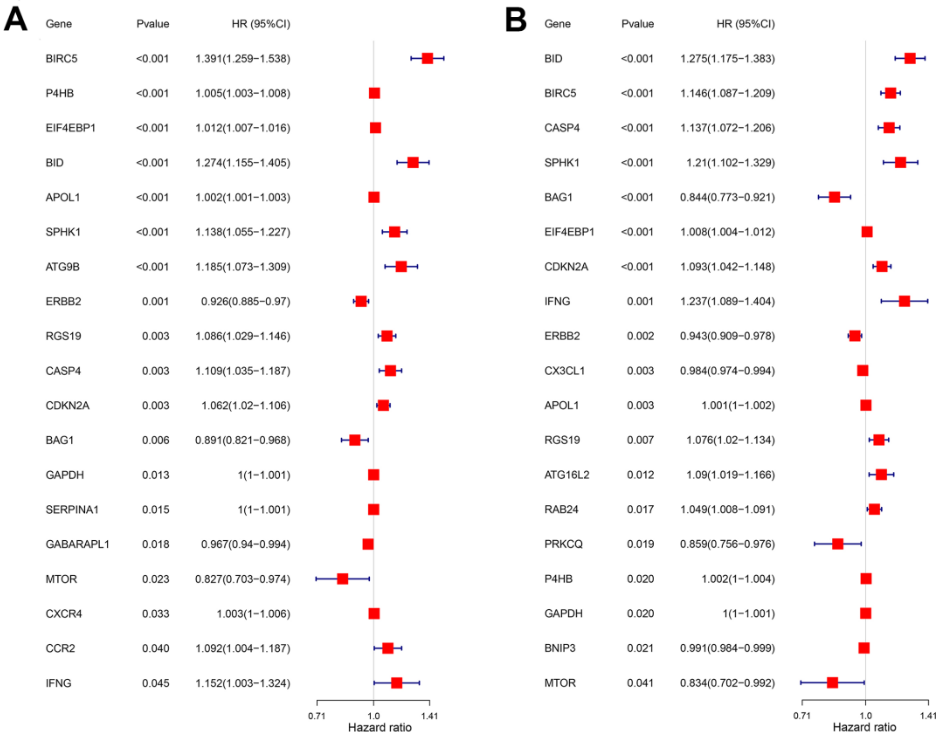

Supplementary Figure 1 - Univariate Cox regression analyses of DEARGs with prognostic potential in the training group ccRCC patients. (**A**) Correlation analysis of DEARGs using the OS model. (**B**) Correlation analysis of using the DFS model. Genes with p-values less than 0.05 are considered prognostic genes.

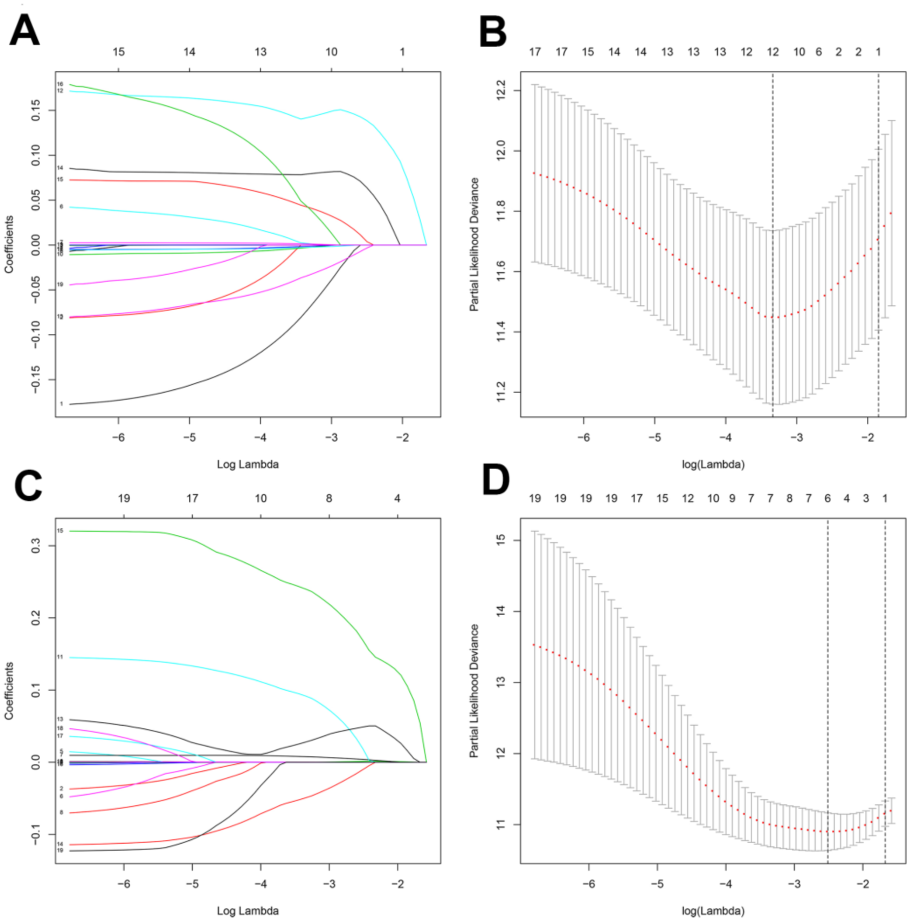

Supplementary Figure 2 - Lasso regression analysis of DEARGs with prognostic potential in the training group ccRCC patients. (**A**–**B**) Lasso regression analyses of DEARGs using the OS model. (**C**–**D**) Lasso regression analyses of DEARGs using the DFS model.
